# Fermented Radish and Beetroot Powder as Natural Sources of Nitrite in Beef Burger

**DOI:** 10.3390/foods15101651

**Published:** 2026-05-09

**Authors:** Samar A. Almohaimeed, Fahad Y. Al Juhaimi, Elfadil E. Babiker, Isam A. Mohamed Ahmed

**Affiliations:** Department of Food Science and Nutrition, College of Food and Agricultural Sciences, King Saud University, Riyadh 11451, Saudi Arabia; 443203878@student.ksu.edu.sa (S.A.A.); faljuhaimi@ksu.edu.sa (F.Y.A.J.); ebabiker.c@ksu.edu.sa (E.E.B.)

**Keywords:** beef burger, fermented radish, fermented beetroot, natural nitrate source, residual nitrite

## Abstract

Growing concern over synthetic nitrite in processed meat has increased interest in natural curing alternatives for clean-label meat products. This research aimed to evaluate the potential of fermented radish and beetroot powders as natural sources of nitrite and bioactive compounds for preserving and improving the quality attributes of beef burgers during refrigerated storage. Burgers were formulated with different levels (3% and 6%) of fermented radish (R3 and R6) and beetroot (B3 and B6) and compared with negative (no additive) and positive (sodium nitrite) controls during 14 days of refrigerated storage, with physicochemical, color, oxidative, microbial, and sensory properties evaluated throughout storage. At day 7, radish and beetroot treatments improved color attributes, reduced lipid oxidation, and controlled microbial growth compared with the negative control. Among the natural treatments, R3 showed the best overall performance, with marked phenolic enrichment, high sensory scores, improved color properties, reduced lipid oxidation, enhanced DPPH radical scavenging activity, and more controlled microbial growth. Radish and beetroot treatments showed performance close to that of the positive control across most evaluated parameters, while showing higher cooking yield, higher phenolic content, greater DPPH radical scavenging activity, better consumer sensory acceptance, and lower diameter shrinkage than the positive control. Overall, the results support the potential of radish and beetroot fermented powders, especially R3, as promising clean-label alternatives for enhancing the quality and storage stability of raw beef burgers.

## 1. Introduction

Red meat remains an important component of the Saudi food supply, with official Saudi statistics reporting a per capita availability of 13.2 kg/year and a red-meat self-sufficiency rate of 62% in 2024 [1]. Burgers are highly favored by consumers due to their affordability, convenience, and quick preparation [2]. They represent a vast economic sector; for instance, the United States alone consumes approximately 5 billion beef burgers annually [3]. In Saudi Arabia, beef or chicken burgers were the most consumed fast-food meals [4]. Although burgers are often categorized as fast food, they remain a nutritionally relevant food vehicle because they can provide high-quality protein, essential vitamins, and minerals, thereby contributing meaningfully to dietary energy and nutrient intake [5].

Nitrite is a key curing agent in processed meats because it contributes to the characteristic cured color and flavor, improves oxidative stability, and enhances microbial safety [6,7]. In meat systems, nitrite is reduced to nitric oxide, which binds to myoglobin to form nitrosomyoglobin and, after heating, nitrosohemochrome, the stable pigment responsible for the typical pink-red color of cooked cured meats, is formed [8]. Nitrite also acts as an effective antioxidant by stabilizing heme iron and limiting metal-catalyzed oxidation, thereby reducing the development of rancidity during storage [7]. In addition, it plays an important antimicrobial role, particularly in inhibiting *Clostridium botulinum*, while also contributing to a broader control of foodborne microorganisms [6,9]. The International Agency for Research on Cancer (IARC) has classified processed meat as carcinogenic to humans (Group 1), and this classification reflects the strength of the evidence for carcinogenicity rather than the magnitude of risk [10]. This carcinogenic potential should not be attributed to added nitrite alone, because several mechanisms have been proposed, including haem iron-promoted endogenous formation of N-nitroso compounds, exposure to exogenous N-nitroso compounds, and the generation of heterocyclic amines and polycyclic aromatic hydrocarbons during high-temperature cooking [10,11]. Nitrite-related risk appears to depend strongly on the food matrix. In processed meats, nitrite may favor N-nitroso compound formation because the meat matrix provides nitrosatable precursors and heme iron, whereas in vegetables, antioxidants and polyphenols can inhibit nitrosation and promote nitric oxide formation instead [11,12]. Global authorities, including WHO through JECFA, have established an acceptable daily intake (ADI) for nitrite of 0–0.07 mg/kg body weight per day [13,14]. In parallel, United States regulations impose strict maximum limits on ingoing synthetic nitrite in processed meats, up to 156 ppm in comminuted cured products [15]. These regulatory restrictions, along with increasing consumer demand for clean-label meat products free of synthetic curing additives, have driven greater interest in natural preservation strategies. As a result, growing attention is being directed toward plant-based curing agents and alternative processing technologies that can preserve product safety and quality while reducing dependence on synthetic nitrite [11,12,15]. The reduction or replacement of synthetic nitrite remains a major technological challenge in processed meats because nitrite simultaneously contributes to the development of characteristic cured color and flavor, oxidative stability, and antimicrobial protection. Despite numerous studies, no single natural alternative has yet been able to comprehensively reproduce all of these functional properties without compromising product quality [15,16].

The use of natural compounds, especially vegetables, as nitrite sources in meat products has gained greater attention in recent decades. Radish and beetroot have been gaining increasing attention in the food industry owing to their effective biological activity. These natural products contain bioactive compounds such as nitrates and phenolic compounds, which serve as preservatives and antioxidants [17,18]. Both radish powder and beetroot powder are rich sources of nitrate, with reported concentrations ranging from 7100 to 55,935 mg/kg and from 3312 to 42,415.16 mg/kg, respectively [19,20,21]. Nitrate levels in radish and beetroot vary due to genetic differences, environmental factors, and agricultural practices [22]. Due to their high nitrate content, beetroot and radish are considered promising natural alternatives to synthetic nitrates in meat products [18]. Furthermore, Saudi Arabia provides a relevant official context for developing locally sourced clean-label meat ingredients from vegetable crops. In 2024, open-field vegetable production reached 2.745 million tons from 89.7 thousand hectares, while protected vegetable production reached 797 thousand tons from 7.8 thousand hectares, supported by more than 121 thousand vegetable greenhouses [23]. Official Saudi sources also identify radish as a winter vegetable crop and include both radish and beetroot among the Kingdom’s cultivated seasonal crops [24,25]. These indicators support the local relevance of radish- and beetroot-based ingredients for the development of clean-label meat products.

Vegetables can serve as effective natural sources of nitrite in meat products after nitrate, the main form found in vegetables and fruits, is converted to nitrite by various methods. Bacterial reduction in nitrate to nitrite is the main process that involves introducing a starter culture of nitrate-reducing bacteria, such as *Staphylococcus carnosus*, to the vegetable powder prior to or during incorporation of the powders into meat products [26]. *S. carnosus* is frequently selected for its ability to perform well under relatively mild fermentation conditions [26]. Radish and beetroot can serve as natural alternatives to nitrite in beef burgers, offering significant promise for the meat industry. These natural components can enhance the reputation of meat products by offering a healthier alternative to synthetic nitrites. Although, both natural and synthetic curing systems rely on nitrite-derived nitric oxide reactions within the meat matrix [27]. The interest in radish and beetroot as natural curing ingredients is more appropriately linked to their clean-label character, consumer demand for naturally derived ingredients, the possibility of lower residual nitrite levels in some pre-converted vegetable-based systems, and their accompanying bioactive compounds [8,27]. The accompanying antioxidant compounds in vegetable-based curing ingredients may help limit nitrosation reactions, particularly when residual nitrite levels are reduced [27]. Additionally, they can improve the nutritional value and potential health benefits of burgers through their bioactive compounds. Incorporating radish and beetroot as natural alternatives to nitrite may enable meat manufacturers to meet consumer demand for clean-label products while ensuring safety and quality [18,27].

Although radish and beetroot powders have shown potential as natural sources of nitrate and bioactive compounds, their application in meat products, particularly fresh meat systems, remains limited [2,6]. Therefore, this study investigated the potential of fermented radish and beetroot powders as natural alternatives to synthetic nitrite and as sources of bioactive compounds to improve and maintain the physicochemical, microbial, and sensory quality attributes of beef burgers during chilled storage.

## 2. Materials and Methods

### 2.1. Materials

Boneless beef (*Bos taurus*) meat of young male beef, in addition to beef back fat of the same animal, was purchased from a slaughtering house (Riyadh, Kingdom of Saudi Arabia) within 1 h of slaughter and was transferred to our laboratory under controlled cold conditions. The fresh radishes, beetroots, and spices were acquired from a local market in Riyadh, Saudi Arabia, and transferred to the laboratory under cold conditions. All chemicals were of analytical grade.

### 2.2. Production of Fermented Radish and Beetroot Powder

Radish and beetroot were prepared by the method designated by Hwang et al. [28], with some modifications. They were carefully washed with water, and then the inedible parts were removed. The pulp was cut into small cubes, freeze-dried, and then milled into fine powder. After that, 10 g of radish and beetroot powder were combined with 100 mL of ddH2O for 30 min. Additionally, the mixture was inoculated with 0.025% of *Lactobacillus sakei* and *Staphylococcus carnosus* starter culture (F-RM-52, Bactoferm^TM^, Chr. Hansen Inc., Milwaukee, WI, USA) and then subjected to 24 h incubation under shaking conditions at 30 °C. The mixture was concentrated to reduce moisture using a vacuum oven at 60 °C (Memmert GmbH + Co. KG, model VO49, Schwabach, Germany) and then kept at 4 °C until utilization in a burger. The fermented vegetable mixture was added to the meat batter at a level equivalent to 3% and 6% of the original vegetable powder used in the fermented samples preparation.

### 2.3. Production of Beef Burgers

The beef burgers were manufactured following the method described by Hawashin et al. [29]. Beef round was used as the lean meat source, and the visible fat and connective tissues were removed before processing. Beef fat was minced separately under the same conditions and added at 9% in all formulations. Lean meat and beef fat were each minced once for 2 min through an 8 mm grinder plate using a Kenwood kitchen mincer (Kenwood Co., Ltd., Havant, UK). Processing was carried out under chilled conditions in an air-conditioned room to minimize temperature rise, and cold water was used in all formulations. After mincing, the meat was immediately covered and transferred to a sanitized refrigerator used exclusively for the samples. During mixing, treatment-specific preparation, portioning, and forming, the batter was kept chilled by returning it to refrigeration between processing steps, with the material covered during each holding period.

Six different formulations of beef burger of 626 g each were prepared by merging minced beef meat with the following: NC (negative control, burgers without sodium nitrite), PC (positive control, burgers formulated with sodium nitrite), R3 (burgers formulated with 3% fermented radish powder), R6 (burgers formulated with 6% fermented radish powder), B3 (burgers formulated with 3% fermented beetroot powder), B6 (burgers formulated with 6% fermented beetroot powder), and spices added according to proportions detailed in Table 1. In the PC treatment, a commercial curing premix composed of 6.25% sodium nitrite and 93.75% NaCl was added at 0.25% of the formulation, corresponding to an ingoing sodium nitrite level of 156 mg/kg product, consistent with the commonly applied U.S. curing system for comminuted cured meat products [30,31]. In the vegetable-treated groups, fermented beetroot or radish mixture powder was incorporated at 3% or 6%, with a corresponding reduction in added cold water, as shown in Table 1. The 3% and 6% addition levels were selected based on a combination of small-scale preliminary burger trials and previous meat-product literature. In a preliminary screening trial, beef burgers formulated with 4% radish and beetroot preparations confirmed the feasibility of incorporating these vegetable materials into the burger matrix and showed clear differences in color and sensory acceptability among treatments. In addition, previous studies on natural nitrite sources in meat products have used vegetable-derived ingredients at levels ranging from less than 1% up to 5%, depending on the ingredient form and meat system, whereas burger and patty studies using plant powders as functional ingredients have frequently evaluated concentrations within the 2% to 6% range [18,28,29,32]. Therefore, 3% was selected as a moderate inclusion level, whereas 6% was selected as a higher but still technologically workable level to evaluate concentration-dependent effects without using excessively high additions likely to impair product quality.

The burger batter was prepared by first mixing minced lean meat, minced beef fat, salt, white pepper, black pepper, garlic powder, onion powder, and sodium ascorbate using a Stephan UM 12 (Stephan U. Sohner GmbH & Co., Hameln, Germany) at 1420 rpm until a homogeneous mixture was obtained. A single common base mixture was first prepared and then divided into six equal portions. Each portion was subsequently mixed separately with cold water and its respective treatment ingredient, namely no curing ingredient for NC, curing mixture for PC, or fermented beetroot or radish mixture powder for the vegetable-treated groups. A burger-forming device (Expro. Co., Shanghai, China) was used to shape burgers (25 g each). Burgers were approximately 70 mm in diameter and 7 mm in thickness.

For cooking the burgers, a convection oven (Hobart Corp., Troy, OH, USA) was used at 160 °C, and a cooked burger was obtained when the central temperature of the burger reached 80 °C, approximately 20 min duration, with flipping the patty frequently every 5 min. For storage stability studies, raw burger samples from each treatment were individually packed in food-grade polyethylene zipper bags under non-vacuum conditions and kept at 4 ± 1 °C in a refrigerator. Analyses were performed at days 0, 7, and 14, with day 0 representing the initial evaluation point on the same day of manufacture. Analytical measurements were performed in triplicate. According to the Saudi technical regulation SFDA.FD 150-1/2018 [33], the required shelf life of refrigerated burger meat packed in suitable containers is 5 days under chilled storage conditions. In this study, day 7 was considered a near-end practical comparison point for refrigerated burger quality, whereas day 14 represented an extended storage challenge to evaluate treatment-dependent changes beyond the typical commercial chilled storage period.

### 2.4. Determination of Nitrate and Nitrite in Radish and Beetroot by Ion Chromatography

Nitrate and nitrite in radish and beetroot powders and their pre-converted mixtures were determined by ion chromatography following the validated anion-exchange IC approach described by McMullen et al. [34], with minor sample preparation modifications to fit the study matrices. Briefly, 0.250 g of the sample was quantitatively transferred into a 100 mL volumetric flask and extracted with nitrate-free deionized water. The mixture was sonicated in an ultrasonic water bath at 37°C for 20 min, cooled to room temperature, and filtered through a syringe membrane filter prior to analysis. Aliquots of the primary extracts were further diluted with deionized water as needed to fall within the calibration range (radish extract, 5.0 mL aliquot; beetroot extract, 2.5 mL aliquot). IC analysis was performed on a Dionex ICS-6000 system (Thermo Fisher Scientific, Waltham, MA, USA) using an anion-exchange analytical column (IonPac AS19-4 um RFIC, Thermo Scientific, Sunnyvale, CA, USA, 250 × 4 mm) with a matching guard column (IonPac AG19 RFIC, Thermo Scientific, Sunnyvale, CA, USA, 50 × 4 mm). Separation was carried out using KOH eluent (Thermo Scientific, Sunnyvale, CA, USA) with a gradient program at a flow rate of 4.0 mL/min, and detection was conducted by conductivity detection (cell heater set at 35 °C). Quantification was performed using external calibration prepared from a certified mixed anion standard (Dionex Seven Anion Standard II) between 1 and 50 ppm (1, 5, 10, 20, 40, 50 ppm). Peak identity was confirmed using retention times from the mixed standard (nitrite RT 7.021 min, nitrate RT 9.197 min). Final concentrations were processed in Chromeleon with sample weight and dilution factors applied within the processing method and reported as mg/kg sample.

### 2.5. Residual Nitrite Analysis

Residual nitrite content in beef burgers was determined according to Merino [35] using the Griess reaction. Briefly, nitrite was diazotized with sulfanilamide and coupled with N-(1-naphthyl) ethylenediamine dihydrochloride to form an azo dye, and absorbance was measured at 540 nm. The calibration curve was prepared from a NaNO_2_ standard solution at 0, 0.2, 0.4, 0.6, 0.8, and 1.2 mg NO_2_^−^/L. Residual nitrite concentration was calculated from the calibration curve equation, corrected for dilution, and expressed as mg NO_2_^−^/kg sample.

### 2.6. Phenolic Content (GAE) Analysis by HPLC-UV

Phenolic content was assessed by HPLC with UV detection at 280 nm and expressed as gallic acid equivalents (GAE), following Tapia-Quirós et al. [36] approach with slight modifications. Beef burger samples (5.0 g) were extracted with 50 mL distilled water: acetone (1:1, *v*/*v*) using an ultrasonic bath for 35 min at room temperature, then centrifuged (5000× *g*). The supernatant was filtered through a 0.22 um syringe filter prior to injection. Chromatographic analysis was performed using a Thermo Scientific Dionex UltiMate 3000 HPLC system (Chromeleon) on a Nucleodur C18 Gravity column (Thermo Scientific, Sunnyvale, CA, USA). The mobile phase consisted of solvent A (water acidified with formic acid, pH 2.6 adjusted with acetic acid) and solvent B (acetonitrile), with isocratic elution (70% A, 30% B) at 0.5 mL/min for 10 min. The injection volume was 10 µL, and the column and autosampler were maintained at 25 C. Quantification was performed using an external gallic acid calibration curve (R2 = 0.9998). Results were expressed as mg gallic acid equivalents (GAE) per kg sample (mg GAE/kg), based on a single integrated marker peak at 280 nm.

### 2.7. Analysis of Antioxidant Activity of Samples (DPPH)

The extracts’ antioxidant activity was assessed by measuring 1,1-diphenyl 2-picrylhydrazyl (DPPH) radical scavenging activity. The method of Singh et al. [37] was applied for the analysis of DPPH radical scavenging activity of radish and beetroot powder, fermented vegetable mixture powder, and meat product samples. In brief, 200 μL of the extracts of samples was diluted with 800 μL of 0.1 M Tris–HCl buffer (pH 7.4). One milliliter of DPPH (250 μM) was added to this mixture and subjected to a vortex mixing process. After keeping the tubes for 20 min in the dark at room temperature, the absorbance was recorded at 517 nm, and DPPH radical scavenging activity was calculated as follows:

(1) $$DPPH\;scavenging\;activity\left(\%\right)\;=\;\frac{A\;blank-A\;sample}{A\;blank}\;\times 100$$

### 2.8. Analysis of Thiobarbituric Acid Reactive Substances (TBARS)

The method stated by Rosmini et al. [38] was applied for measuring lipid oxidation of beef burgers. Briefly, a fresh beef burger sample (5.0 g) was homogenized with distilled water (20 mL), and the homogenate was sonicated for 10 min, then filtered through Whatman No. 1 filter paper. An aliquot (1 mL) of the filtrate was transferred into screw-cap tubes, followed by adding 4 mL of 20% thiobarbituric acid and 100 μL of 10% butylated hydroxytoluene. The mixtures were subjected to 10 min incubation in a boiling water bath at 100 °C for developing the pink color, and then the tubes were cooled down using running tap water. After centrifugation (5500× *g*, 25 min), the supernatants were collected, and their absorbance was recorded at 532 nm. Quantification was performed using an external calibration curve prepared from malondialdehyde tetrabutylammonium salt, and concentrations were obtained from the regression equation of the calibration curve. Results were expressed as mg MDA equivalents/kg sample after applying a molecular weight correction factor (72.06/313.52 = 0.2298). Triplicate samples were analyzed.

### 2.9. Color Measurement

The surface color attributes [lightness (L*), redness (a*), and yellowness (b*)] of raw non-formulated and formulated beef burgers were assessed by a Hunter Lab colorimeter (Miniscan^®^ XE plus 4500L, Reston, VA, USA) as described in the instruction manual. The colorimeter was calibrated using a standard white plate before the color attributes were analyzed. Three measurements were taken for each sample at three different locations in the sample.

### 2.10. Microbiological Analyses of Meat Products

The standard method stated by the International Commission on Microbiological Specification for Foods [39] was applied for the analysis of the total viable count (TVC) of beef burgers during storage. In short, 9 milliliters of sterile peptone water (0.1% sterile) was used to homogenize a 1 g meat product. After that, 10-fold serial dilutions of 1 mL of the meat samples’ homogenates in 9 mL of 0.1% peptone water were prepared. Following that, the pour plating method was used to plate 1 mL of suitable serial dilutions onto plate count agar and after 48 h incubation at 37 °C the colonies were counted and the results were specified as log10 cfu/g. In all samples, duplicate plates of appropriate serial dilution were used.

### 2.11. Measurement of pH

The pH of the beef burger samples was determined using a portable meat pH meter (Model HI99163, Hanna Instruments, Woonsocket, RI, USA) with a penetrating electrode [40]. Prior to measurements, the pH meter was calibrated using standard buffer solutions at pH 4.01 and 7.01. The probe was inserted directly into the burger patties at three different locations to ensure representative readings. For the pH of radish and beetroot powder, and fermented vegetable powders, 1 g of the samples in 10 mL distilled water was measured using a pH meter probe (Corning Scientific Products, New York, NY, USA).

### 2.12. Determination of Chemical Composition

The official standard methods [41] were used to assess the moisture (Method No. 940.09), fat (Method No. 960.39), protein (Method No. 992.15), and ash (Method No. 942.05) composition of raw beef burgers.

### 2.13. Evaluation of the Cooking Characteristics of Beef Burgers

The cooking characteristics, including cooking yield and diameter changes, were evaluated by applying the methods and formulas reported previously [32]. The cooking yield percentages were determined by dividing the cooked burger weight by that of the raw ones, as mentioned in Murphy et al. [42] method. The diameter shrinkage was determined using the equations described by Murphy et al. [42].

(2) $$\begin{array}{c} Diameter\;shrinkage\left(\%\right)\\ =\;\frac{\left(Raw\;thickness-Cooked\;thickness\right)+(Raw\;diameter-Cooked\;diameter)}{Raw\;thickness+Raw\;diameter}\times 100 \end{array}$$

### 2.14. Texture Profile Analysis (TPA) of Meat Products

For measuring TPA of raw, fresh, and stored beef burgers, a Brookfield texture analyzer (Model CT3, Brookfield, Middleboro, MA, USA) was used as stated earlier [43]. A 2-cycle set was applied to analyze the TPA parameters, namely hardness (kg), cohesiveness, springiness (mm), and chewiness (hardness × cohesiveness × springiness) in triplicate analyses of all samples.

### 2.15. Sensory Analysis

Sensory evaluation of cooked beef burgers was performed at day 0, 7, and 14 of refrigerated storage. Fifty semi-trained panelists aged between 20 and 40 years evaluated the samples using a 9-point hedonic scale, where 1 = extremely dislike and 9 = extremely like. The evaluated sensory attributes were color, appearance, taste, texture, flavor, tenderness, juiciness, and overall acceptability. At each storage time, each panelist evaluated all six burger formulations. Samples were coded with random three-digit numbers and presented in a randomized order, and water was provided between samples for palate cleaning. The same panelists participated in the evaluations at all storage times. Individual panelist scores were used in the statistical analysis, and mean panel scores are presented in the table. The scores of means ≥ 5 were considered satisfactory [44]. Prior to the sensory analysis, all participants gave written informed consent, and the analysis received approval from the King Saud University Ethics Committee (IRB Approval of Research Project No E-25-10195).

### 2.16. Statistical Analysis

Data were analyzed using XLSTAT 2019 (Addinsoft, New York, NY, USA). The effects of treatment, storage time, and their interaction were evaluated by two-way analysis of variance (ANOVA). When significant differences were detected, means were separated using Duncan’s multiple range test. Results were expressed as mean ± standard error (SE), and significance was declared at *p* < 0.05. For sensory analysis, individual panelist scores were used in the statistical analysis, whereas mean panel scores are presented in the tables.

## 3. Results and Discussion

### 3.1. Nitrate and Nitrite Content, pH, DPPH in Radish and Beetroot Powders and Their Fermented Mixtures

Table 2 shows clear differences in nitrate concentration, nitrite concentration, pH, and antioxidant activity between beetroot and radish powders before and after fermentation. Radish powder showed a higher nitrate concentration (25,623.6 mg/kg) than beetroot powder (3805.42 mg/kg). The higher initial nitrate concentration observed in radish powder than in beetroot powder agrees with previous reports. Ozaki et al. [18] found that radish powder contained 16,263.87 mg/kg nitrate, compared with 14,037.82 mg/kg in beetroot powder. A similar trend was reported by Kassem et al. [19], who also found higher nitrate content in radish powder (7100 mg/kg) than in beetroot powder (3312 mg/kg), supporting the same pattern observed in the present study. After fermentation or nitrate-to-nitrite pre-conversion, nitrate content decreased in both, reaching 1588.72 mg/kg in fermented radish and 351.06 mg/kg in fermented beetroot, corresponding to reductions of approximately 93.8% and 90.8%, respectively. In contrast, nitrite was reported in the fermented samples, with a higher value in fermented beetroot (751.99 mg/kg) than in fermented radish (389.11 mg/kg).

Fermentation was also associated with a noticeable decline in pH, decreasing from 6.56 to 4.40 in beetroot and from 6.16 to 3.81 in radish. These findings are consistent with previous studies on pre-converted vegetable nitrite sources. Choi et al. [45] reported that fermented red beet produced using *Staphylococcus carnosus* reached a nitrite concentration of 729.28 ppm with a final pH of 4.65. Similarly, fermented red beetroot showed a pH of 4.56 and a nitrite concentration of 748.51 ppm [28]. Moreover, the previous literature indicates that final nitrite yield is determined not only by the initial nitrate concentration, but also by the efficiency of microbial nitrate reduction during fermentation. For example, one study showed that although young radish contained more than five times the initial nitrate level of a commercial vegetable powder (3931 mg/kg vs. 740 mg/kg), both substrates produced nearly similar amounts of nitrite after 24 h of incubation (68 mg/kg vs. 54 mg/kg) [46]. This study supports the present results and suggests that nitrite after fermentation is affected by conversion efficiency and fermentation conditions more than by the initial nitrate levels.

Regarding antioxidant activity, fermented beetroot showed slightly higher DPPH inhibition (67.56%) than fermented radish (63.36%). Research on beetroot powder reported a DPPH free radical scavenging activity of 65% inhibition, while another study evaluating beetroot pulp recorded a DPPH activity of 70% inhibition [6]. In contrast, most studies report radish DPPH in Trolox equivalents rather than percentages. Direct comparisons between the two vegetables confirm the trend. A study evaluating both powders found that beetroot exhibited much higher DPPH radical scavenging activity (7140.00 mg Trolox/g) compared to radish powder (1537.65 mg Trolox/g) [18]. The higher DPPH scavenging activity in beetroot may be related to the antioxidant compounds naturally present in beetroot, particularly betalains such as betanin, as reported in previous studies [6]. Overall, radish powder had higher nitrate content than beetroot powder, whereas fermented beetroot showed higher nitrite concentration and slightly higher DPPH scavenging activity than fermented radish.

### 3.2. Residual Nitrite Contents

Table 3 shows that residual nitrite was significantly affected by treatment, storage, and their interaction (*p* < 0.01). As expected, PC showed the highest residual nitrite at all storage times, whereas NC showed the lowest values overall. The low residual nitrite detected in NC, despite the absence of intentionally added nitrite, is likely to originate from trace nitrate or nitrite introduced with minor formulation ingredients, especially spices and other dry seasonings, and possibly water. Since nitrite was already detected at day 0, ingredient-derived background input appears more likely than formation during storage alone [47]. All natural treatments showed residual nitrite levels higher than NC and lower than PC, indicating measurable nitrite contribution from the fermented vegetable systems, but at lower levels than synthetic nitrite. Hwang et al. [48] evaluated pre-converted nitrites from red beet, spinach, celery, and lettuce in pork sausages. They found that the residual nitrite contents in all treatments were significantly lower than the positive Control (150 ppm synthetic nitrite) but remained at higher levels than the negative Control (nitrite-free).

The residual nitrite of PC at day 0 was 32.48 mg/kg, despite the added nitrite amount being 156 mg/kg. The literature shows that the residual nitrite content measured in the final meat product is significantly lower than the initial amount of nitrite added, regardless of whether it is derived from natural sources (like fermented radish or beetroot) or added as a synthetic material (like sodium nitrite) [21]. According to Xi et al. [49], the residual nitrite amount was about 75% of the initial concentration after the production process and ranged between 4 mg/kg and 10 mg/kg at the end of the storage period. Another study found that immediately after adding 150 mg/kg of synthetic sodium nitrite to a raw meat batter, only 93.15 mg/kg (about 62.1%) could be detected due to its immediate reaction with the meat and exposure to light, oxygen, and ambient temperatures [18]. Nitrite is a highly reactive molecule; once added to a meat system, it immediately begins to react with or bind to various chemical components. It reacts with myoglobin to form the pink cured meat pigment (nitrosylmyoglobin), binds to non-heme proteins, and reacts with lipids to prevent oxidation. Because it is actively being consumed to cure the meat, the analytically detectable amount drops significantly [21]. Also, because the reducing agent sodium ascorbate is present in the formulation, it actively accelerates the conversion of nitrite to nitric oxide to speed up color development, which further drives down the residual nitrite levels [21,45].

Across storage, different patterns of residual nitrite were observed among treatments. Residual nitrite in PC and R6 decreased continuously during storage, whereas R3, B3, and B6 decreased at day 7 and then increased again by day 14. This pattern may reflect differences between direct synthetic nitrite addition and fermented vegetable-based nitrite systems. Synthetic nitrite is often reported to decline more consistently during storage, whereas residual nitrite in products formulated with nitrate-rich vegetable sources may fluctuate depending on nitrate conversion, nitrite reactions, and matrix conditions. Because the methodology we used in this study retained the whole fermented vegetable mixture powder and the starter culture, the natural treatments may have contained a reservoir of unreacted nitrate [26]. During storage, the starter culture bacteria may have continued to gradually reduce part of this remaining nitrate into nitrite, possibly contributing to a secondary increase in detectable nitrite levels by day 14. Furthermore, nitrite can be converted into intermediate nitrogen compounds such as nitric oxide during curing reactions; these components may interact reversibly with meat components over time, which could contribute to fluctuations in the detectable residual nitrite [9,21].

At day 7, all treatments remained significantly lower than PC, but they were higher than NC, confirming partial nitrite retention relative to the untreated control. Among the natural treatments at day 7, R6 showed the highest residual nitrite, while R3 and B3 showed the lowest. By day 14, B3 and B6 showed the highest residual nitrite among the natural treatments and were similar to each other, while R6 showed the lowest value among the natural alternatives. Overall, natural treatments provided measurable residual nitrite compared with NC, with B3 and B6 showing better residual nitrite persistence at the end of storage, although none approached PC. These results confirm that residual nitrite should not be used as a single measure to judge the replacement potential of PC. Because nitrite contributes to multiple quality and safety attributes, replacement potential should be assessed using multiple indicators, rather than residual nitrite alone [9].

### 3.3. Bioactive Properties

Table 3 shows that phenolic content and DPPH radical scavenging activity were significantly affected by treatment, storage, and treatment x storage interaction (*p* < 0.01). Overall, fermented radish and beetroot mixture powder treatments increased phenolic content and DPPH inhibition compared with NC and PC at most storage times, which supports their contribution as sources of bioactive compounds in beef burgers during cold storage.

The phenolic-rich treatments were associated with greater antioxidant protection and overall quality retention throughout the 14-day storage period compared to the controls. The data show that retaining the whole fermented vegetable mixture powder preserved phenolic compounds, which may have contributed to better quality retention during storage. The treatments formulated with the fermented vegetable mixtures maintained significantly higher levels of phenolic contents by the end of storage. By day 14, the R3, R6, and B6 treatments retained high phenolic content (ranging from 300.33 to 328.00 mg GAE/kg), whereas NC and PC decreased to 34.54 and 37.43 mg GAE/kg, respectively.

These higher phenolic values were accompanied by stronger radical-scavenging activity in several treatments, although this pattern did not uniformly correspond to TBARS. At day 14, the B6 treatment showed the highest 76.34% DPPH inhibition, and B3 and R6 also showed relatively high DPPH inhibition (72.90% and 69.85%, respectively), whereas NC showed higher DPPH inhibition than both PC and R3. The relatively higher DPPH activity observed in NC than in PC and R3, despite its much lower phenolic content, suggests that DPPH in meat extracts may reflect not only phenolic compounds but also other extractable reducing substances in the meat matrix, and therefore should not be interpreted as a direct indicator of better oxidative stability [50]. In addition, the lower DPPH response of R3 at day 14, despite its high phenolic content, may reflect reduced extractability and/or altered analytical recovery of phenolic compounds during storage due to their interactions with meat proteins and other matrix components [51]. Thus, the higher DPPH value of NC should be interpreted cautiously, particularly because NC still showed high TBARS at the same storage time [50]. As shown in Table 2, fermented beetroot and fermented radish exhibited DPPH inhibition values of 67.56% and 63.36%, respectively. Beetroot antioxidant activity has been linked to betalains, particularly betanin, together with phenolic acids, whereas radish antioxidant activity has been linked to phenolic compounds, flavonoids, and anthocyanin-related constituents [6,18]. These compounds may contribute through hydrogen atom or electron donation and radical quenching. A study evaluating the addition of beetroot powder to chicken sausages found similar DPPH radical scavenging results to B3 and B6 treatments. The researchers observed that incorporating beetroot powder increased the DPPH radical percentage inhibition in the meat. The inhibition values scaled directly with the amount of beetroot powder added, reaching up to 68.44% at their highest inclusion level [52].

Across storage, different patterns of phenolic content were observed among treatments. NC and PC decreased by day 14, while R3, R6, and B6 maintained much higher phenolic values throughout storage; B3 was high at day 0 but dropped by day 14. As storage progresses, phenolics can be oxidized to quinones and then undergo polymerization or form covalent adducts with nucleophilic amino acid side chains. These reactions reduce the pool of free extractable phenolics, so the measured phenolics can drop even if the compounds are still present in bound or transformed forms [53].

At day 7, R3 showed the highest phenolic content, followed by R6 and B6, and all were higher than PC and NC. In contrast, B3 was the lowest among natural treatments at day 7 and was slightly lower than NC and PC. By day 14, R3, R6, and B6 remained much higher than PC and NC, while B3 decreased to 128.67 but remained above NC and PC. The increase in R3 at day 7 likely reflects improved extractability or release of phenolics, whereas the subsequent decline by day 14 is consistent with the potential of consuming phenolic content to prevent lipid oxidation. At day 7, the higher measured phenolic content in R3 than R6 may indicate that a greater detectable free phenolic fraction remained available at the moderate inclusion level, whereas the higher inclusion level may have promoted phenolic interactions with matrix components. Such interactions can occur between phenolics and food macromolecules, including proteins and carbohydrates, and may affect the measurable or extractable phenolic fraction in complex food systems [53,54]. In addition, protein-phenolic interactions may occur through both non-covalent and covalent mechanisms, and their extent depends on phenolic concentration and system conditions [54]. Under oxidative conditions, some phenolics may also be converted to quinones, which can react with nucleophilic groups in proteins and thereby reduce the detectable free phenolic fraction during storage; similar covalent adduct formation has been demonstrated in myofibrillar protein systems [55,56]. Therefore, the lower phenolic value observed in R6 may reflect greater matrix association and/or oxidative conversion of phenolics during storage rather than a lower intrinsic phenolic potential. Overall, R3 showed the highest phenolic content at mid- and late storage, followed by R6 and B6, suggesting better potential for supporting quality preservation through bioactive compounds during cold storage.

At day 7, B3 showed the highest DPPH activity, followed by R3 and B6, all of which were higher than NC and PC, whereas R6 showed the lowest value. At day 7, R6 showed the highest TBARS value, which was consistent with its lowest DPPH value at the same time point. However, at day 14, R6 increased from 25.57% to 69.85%. This fluctuation was not unique to R6, because B3 and B6 also increased from 53.44% and 46.95% at day 7 to 72.90% and 76.34% at day 14, respectively, while NC rose more slightly from 42.75% to 49.62%. In contrast, PC and R3 showed a continuous decline to 24.81% and 30.15% by day 14, indicating that the DPPH response during storage was treatment-dependent rather than uniform across formulations. A similar mixed pattern was reported by Al-Juhaimi et al. [44], who observed a decrease-then-increase trend in the control and 2% Argel leaf powder (ALP) camel patties, whereas the higher ALP levels (4 and 6%) showed a continuous decline during storage. The authors linked the initial reduction to the utilization and hydrolysis of antioxidant compounds during storage [44]. Related treatment-dependent changes were also reported by Jung et al. [57], where DPPH activity increased in onion peel extract treatments but decreased in the control and onion extract treatment during storage.

By day 14, B6, B3, and R6 showed the highest antioxidant activity and were clearly higher than NC, while PC remained the lowest. However, higher DPPH inhibition did not always correspond to lower TBARS, as R6 combined high DPPH activity with the highest TBARS value at day 14. Overall, B3, R3, and B6 showed good antioxidant enhancement and persistence during cold storage. Al-Juhaimi et al. [44] noted that the DPPH values of the treated patties were higher than those of the untreated ones throughout the storage period.

Across storage, PC showed a continuous decline in DPPH inhibition, while the natural treatments followed different patterns, where R3 decreased over time, R6 dropped at day 7 then increased by day 14, and B3 and B6 increased by day 14. NC also increased from 42.37% at day 0 to 49.62% at day 14, indicating that storage-related changes in extractable radical-scavenging compounds were not limited to the vegetable-treated samples. This increase in NC may be explained by changes occurring within the meat matrix during refrigerated storage. Although NC did not contain added vegetable-derived antioxidants, DPPH is an assay-specific measure of radical-scavenging capacity in the extractable fraction and should not be interpreted as a direct measure of lipid oxidative stability in the meat product [50]. In addition, meat proteins can be a source of antioxidant peptides after proteolysis, and these low-molecular-weight peptides may contribute to radical-scavenging activity through hydrogen atoms or electrons [58]. Therefore, the higher DPPH inhibition in NC at day 14 likely reflects increased extractability or formation of meat-derived reducing compounds rather than improved lipid oxidative stability. This interpretation is supported by the TBARS results, where NC still showed a high TBARS value at day 14 (2.19 mg MDA/kg) despite its increased DPPH inhibition. The PC showing a continuous decline in DPPH % inhibition is consistent with the progressive consumption of nitrite-derived antioxidant activity during storage. Synthetic sodium nitrite does not remain stable in meat; it is highly reactive and becomes consumed as it performs its functions [59]. Therefore, the decline of DPPH inhibition in PC is more cautiously interpreted as a decrease in the extractable reducing capacity associated with nitrite-related reactions during storage, rather than as a direct measure of lipid oxidation protection [50,59]. Meat is a highly oxidative environment, especially when ground/minced and during refrigerated storage, where the oxidation process is continued. Any antioxidant present in the meat, whether it is synthetic or natural nitrite and/or a natural phenolic, is continuously consumed to inhibit free radical chain reactions. However, the DPPH response may also change because antioxidants and other reducing compounds can become less extractable or chemically transformed during storage, especially in complex meat matrices [50]. At the same time, the increase in DPPH activity in NC and some treatments at day 14 may reflect storage-related changes in extractability and/or concentration effects during storage rather than improved overall oxidative stability. For the natural treatments that increased at day 14, especially R6, B3, and B6, the late increase may additionally reflect the release, transformation, or altered extractability of plant-derived reducing compounds from the fermented radish and beetroot matrices during storage. This explanation is supported by studies showing that phenolic compounds can interact with proteins through non-covalent and covalent mechanisms, which can alter the measurable phenolic fraction and antioxidant response in food matrices [51,54]. However, this response was not necessarily linked with lower TBARS, as R6 showed high DPPH inhibition at day 14 while also recording the highest TBARS value. Thus, DPPH should be interpreted as an indicator of extractable radical-scavenging capacity, whereas TBARS reflects lipid oxidation products formed within the meat matrix [50].

### 3.4. Lipid Oxidation (TBARs)

Table 3 shows that TBARS values were significantly affected by treatment, storage, and treatment x storage interaction (*p* < 0.01). Overall, PC showed the lowest lipid oxidation at day 0 and day 7, whereas by day 14, B6 showed the lowest TBARS value. Thus, PC provided the greatest overall oxidative stability during early and mid-storage, while some natural treatments, particularly B6 and to a lesser extent R3 and B3, showed improved late-stage oxidative stability.

Across refrigerated storage, TBARS showed treatment-dependent temporal changes rather than a uniform pattern. NC, R3, R6, and B3 increased from day 0 to day 7, indicating an early rise in lipid oxidation, whereas PC and B6 decreased over the same period. By day 14, PC increased again, while NC, R3, R6, and B3 declined from their day 7 values, although NC and R6 remained among the highest TBARS treatments. Notably, B6 was the only treatment that showed a continuous decrease throughout storage, declining from 1.98 mg MDA equivalents/kg at day 0 to 1.63 at day 7 and 0.89 at day 14. The increase-and decrease pattern in TBARS during storage is reasonable because malondialdehyde and other secondary oxidation products can continue reacting with proteins and other matrix components, which may alter the amount of oxidation products detectable by the TBARS assay during storage [60,61]. Therefore, a later decrease in measured TBARS should not necessarily be interpreted as reversal of oxidation, but rather as dynamic behavior of secondary oxidation products within the meat system [60,61]. The consistently high TBARS in R6, despite higher phenolic content and greater DPPH scavenging at day 14, should be interpreted cautiously, because phenolic and DPPH assays mainly reflect assay-specific antioxidant capacity and do not fully capture antioxidant behavior in a complex meat matrix, whereas TBARS reflects lipid oxidation in the product itself, and no single antioxidant assay captures all aspects of antioxidant behavior in a complex meat matrix [50]. In addition, the higher TBARS in R6 than in R3 suggests that increasing radish addition did not improve oxidative stability in this ground, non-vacuum burger system. Grinding and aerobic storage increase oxygen exposure and accelerate oxidation, while lipid oxidation in meat is strongly promoted by heme proteins and iron, including myoglobin/metmyoglobin-mediated reactions [62]. Under such iron-rich conditions, phenolic compounds may not always act purely as antioxidants and, at higher concentrations or in the presence of transition metals, may even shift toward pro-oxidant behavior [62,63]. Thus, the high TBARS in R6 is better interpreted as a matrix-dependent and dose-dependent response than as direct evidence that radish itself is inherently pro-oxidant. Likewise, plant-based nitrite alternatives do not always provide consistent inhibition of TBARS across different meat systems and storage conditions, as also reported in fermented beef sausage reformulated with beetroot powder as a nitrite alternative [7,21].

At day 7, PC recorded the lowest TBARS, and both B3 and B6 were significantly lower than NC and were also lower than R3 and R6. This indicates that beet-based treatments, B3 and B6, improved oxidative stability relative to NC during mid-storage, while R6 showed the lowest performance. The results showed that treatments B3 and B6 maintained excellent free-radical scavenging activity (DPPH) and effectively suppressed lipid oxidation (TBARS) compared to the controls. This is strongly supported by previous studies demonstrating that the specific bioactive profile of beetroot stands out in meat matrices. Hwang et al. [28] evaluated fermented red beet extracts in low-salt frankfurters and found that by the end of a 4-week refrigerated storage period, the sausages treated with fermented red beet extracts effectively inhibited lipid oxidation, recording significantly lower TBARS values than the untreated controls. The authors attributed this protective effect directly to the stable antioxidant and cyclic amine compounds naturally preserved in the fermented red beet matrix [28]. Another study incorporating beetroot powder into chicken sausages found that beetroot acts as a source of natural antioxidants due to its high concentration of betalains and phenolic compounds. The researchers observed that DPPH radical scavenging activity increased significantly in direct proportion to the amount of beetroot powder added. In practical terms, these results are also relevant because TBARS values higher than 2 mg MDA/kg in beef burgers have been reported to be associated with undesirable sensory changes and consumer perception of oxidation [64]. Accordingly, at day 7, both B3 (1.62 mg MDA/kg) and B6 (1.63 mg MDA/kg) remained below this commonly cited burger-related threshold, whereas NC (2.26 mg MDA/kg) and R6 (2.46 mg MDA/kg) exceeded it. Therefore, the beetroot treatments improved oxidative stability relative to NC and radish treatments at mid-storage and helped maintain TBARS below the commonly cited 2 mg MDA/kg threshold for beef burgers.

By day 14, B6 showed the lowest TBARS, and both R3 and B3 were also low, all lower than NC and R6, and lower than PC. Overall, B6, then R3 and B3, showed better late-stage oxidative stability, although this did not indicate better overall oxidative stability than PC across the entire storage period, because PC remained the most stable treatment at the earlier storage times. Using 2.0 mg MDA/kg as a practical limit associated with acceptable oxidative status in meat products such as fermented sausages and burger-type systems [18,64], NC and R6 exceeded this level at day 7 and/or day 14, whereas PC, R3, B3, and B6 remained below this level at day 14. Therefore, the natural treatments, particularly B6 and R3, may be considered acceptable from the standpoint of late-stage lipid oxidation, but they did not uniformly match PC across all storage times. These results suggest that the bioactive compounds in the beetroot and radish mixtures were associated with lower TBARS in some treatments, especially B6, but this effect was treatment-dependent and did not occur uniformly in all formulations. A study evaluated fermented dry sausages manufactured with radish and beetroot powders stated that all treatments formulated with the vegetable powders showed significantly lower TBARS values than the negative control sausages [18].

Moreover, R6 showed the highest TBARS at day 7, indicating that higher residual nitrite alone was insufficient to prevent lipid oxidation, and that treatment-specific matrix effects and heme-iron-driven pathways likely dominated oxidative outcomes in this formulation. Sucu & Turp [21] found that despite using high concentrations of beetroot powder, the lipid oxidation (TBARS) values of the treated sausages were significantly higher than the synthetic nitrite control during storage. A comprehensive review by Ferysiuk & Wójciak [7] explains that combining nitrites with high doses of complex plant matrices often triggers unexpected lipid oxidation. They highlight multiple studies (e.g., [65] with green tea, and [66] with tomato pulp) where the combination of plant extracts and nitrite created an “antagonistic effect” that acted as a pro-oxidant. However, this inference should not be generalized to all 6% plant treatments, because the same response was not observed in B6. Therefore, the elevated TBARS in R6 is better interpreted as a treatment-specific and matrix-dependent response in the radish formulation under the present burger conditions. One possible explanation is that the radish matrix may have contributed trace transition metals, such as iron or copper, which are known to catalyze lipid oxidation in meat systems; however, this was not measured directly in the present study and therefore remains unproven. Radish has been reported to contain minerals including copper, while lipid oxidation in muscle foods is strongly promoted by transition metals and heme proteins [62,67]. Thus, the high TBARS in R6 may reflect a radish-specific interaction with the burger matrix under aerobic refrigerated storage.

### 3.5. Color Properties

Table 4 shows that L*, a*, and b* were significantly affected by treatment, storage, and treatment x storage interaction (*p* < 0.01). Overall, radish and beetroot treatments modified color development during cold storage, with beetroot treatments showing a darker appearance (lower L*) and radish treatments showing a better retention of redness (higher a*) at later storage. Beetroot contains natural pigments called betalains, including betacyanins and betaxanthins. These pigments are highly concentrated and significantly lower the lightness (L*) of the meat, resulting in a darker and deeper red appearance [18,21]. Ozaki et al. [18] observed that the addition of beetroot powder significantly decreased L* values, resulting in a darker appearance and increased a* values compared to controls. Conversely, treatments formulated with radish powder successfully maintained their color and L* values over 60 days of storage, performing comparably to the synthetic nitrite control. Hwang et al. [28] observed that adding fermented red beet to frankfurters decreased lightness (L*) and increased redness (a*), explicitly noting that the color stability during extended refrigerated storage was successfully maintained by the strong antioxidant properties of the fermented natural extracts. The color of meat products is one of the most important factors considered by consumers when they judge the acceptability of meat products. Discoloration of meat products will have lower consumer acceptability during refrigerated storage, thus causing a major problem for marketing. Therefore, the color stability of meat products can be maintained using antioxidant properties and nitrite from natural sources.

Across storage, L* generally decreased on day 7 and then increased by day 14, but the level depended on formulation. At all time points, B3 and especially B6 showed lower L* than NC and PC, indicating a consistently darker product. At day 7, B6 and B3 were the lowest, while NC and R6 were significantly similar, with the highest values, followed by PC and R3. The L* value confirmed that beetroot treatments were consistently darker, which is expected with beetroot pigments. Although sensory color scores remained high for beetroot treatments across storage, meaning panelists did not reject the darker appearance.

Redness (a*) declined in NC and PC during storage, while several natural treatments maintained higher a* at day 7 and day 14. At day 7, PC showed a higher a* than NC. Both R6 and R3 were closer to PC, where B3 remained the lowest. By day 14, R6 showed the highest a*, followed by R3, B6, and B3, all of which were higher than NC and PC. Standard packaging can affect the color of nitrite-treated samples and directly causes the phenomenon where the surface turns grayish/brown while the interior remains red, and this is what we observed with the PC color. To maintain a cured meat color throughout extended shelf-life, it is generally accepted that a small amount (10–15 ppm) of residual nitrite is needed to serve as a reservoir for the regeneration of cured meat pigment lost from oxidation and light-induced fading [68]. However, the residual nitrite of PC at day 14 decreased to (4.27 mg/kg), which is lower than the amount needed to maintain the redness of cured meat pigment. When the NC and PC faded significantly at day 14, the natural treatments, particularly R6, still maintained a higher a* value. However, R6 showed the highest TBARS value at day 14. This indicates that redness stability and lipid oxidation were not directly linked in this formulation. The higher a* of R6 is more likely linked to color-specific mechanisms, such as cured pigment formation and/or stability within the meat matrix. Similar divergence has been reported in naturally cured meat systems, where vegetable sources such as white kimchi or celery produced cured color comparable to sodium nitrite controls, yet showed higher TBARS values [69]. A comparable pattern was also reported in radish-based reformulated sausages, where adequate color development was achieved while lipid oxidation remained insufficiently controlled during storage [70]. A study supports this observation that phenolic-rich plants actively protect meat pigments during cold storage. Investigating beef burgers formulated with various concentrations of clove powder, the researchers observed a reduction in color properties in the untreated controls during storage. However, the burgers formulated with the phenolic-rich clove powder successfully maintained their a∗ values. The authors attributed this color preservation to the preventive effect of the extract against discoloration [71].

b* tended to decrease at day 7, then partially recover by day 14 in NC and PC, with a decrease during storage for most vegetables’ treatments. b* tended to decrease at day 7, then partially recover by day 14, with clear treatment differences. At day 7, PC showed the lowest b*, while R6 and B6 were the highest, and NC and R3 were intermediate. By day 14, PC and B6 were higher than R3 and B3, suggesting different pigments and storage behavior across formulations.

The higher residual nitrite in R6 at day 7 (Table 2) aligned with improved redness stability, as R6 showed higher a* values than NC and was closer to PC at the same point, supporting the role of nitrite availability in cured color development. Studies confirmed that despite having much lower residual nitrite than the PC, the natural treatments contained sufficient nitrite availability to bind with myoglobin, successfully developing and maintaining the cured redness (a∗) of the meat compared to the fading NC [45].

### 3.6. Microbiological Load and pH

Table 5 shows that total viable counts and pH were significantly affected by treatment, storage, and treatment x storage interaction (*p* < 0.01). Overall, microbial counts increased from day 0 to day 7 in all treatments, then decreased by day 14, with clear treatment-dependent differences during storage. Although a comparable increase-then-decline pattern has been reported in some burger systems subjected to stronger ecological constraints, such as bioprotective cultures, most studies on refrigerated raw beef burgers or beef patties describe a progressive increase in microbial counts during storage, even when added ingredients slow microbial growth [71,72,73,74]. Therefore, the day-14 reduction observed in the present study is more appropriately interpreted as a decline from the day-7 peak in culturable cells, possibly associated with partial oxygen limitation within the zipper bags, storage-related shifts in microbial ecology, metabolite accumulation, and competition for available nutrients during chilled storage, rather than as a complete return to baseline of microbial status [75,76].

At day 0, microbial counts were within a close range across treatments, with PC showing the lowest value, while NC and most natural treatments were the highest. At day 7, B3 showed the lowest counts, followed by PC and R3, all of which were lower than NC, whereas B6 and R6 were closer to NC. Ozaki et al. [18] investigated fermented sausages manufactured with 0.5% and 1% radish and beetroot powders. They found that treatments with these vegetable powders showed high initial counts of coliforms (up to 1100 MPN/g) (most probable number per gram) compared to the synthetic nitrite control. Sucu & Turp [21] similarly observed that bacterial counts were highest in meat formulated with high amounts of beetroot powder. They concluded that introducing higher levels of plant powder may provide a carbohydrate/sugar-rich matrix that can serve as an available nutritional substrate for bacterial growth. B3 showed the lowest microbial count at day 7, which could reflect a combined effect of nitrite chemistry and plant-derived bioactive compounds, rather than a single cause. Although residual nitrite in B3 is lower at day 7, this does not eliminate antimicrobial action because nitrite can be consumed through reactive pathways that generate inhibition, so lower residual values can correspond with antimicrobial activity. In parallel, phenolic compounds can inhibit microbial growth by disrupting cell membranes and stress response systems, and B3, showing the highest DPPH inhibition at day 7, is consistent with an active antioxidant environment that may reduce microbes [77]. The decline in measured phenolics from day 0 to day 7 is also expected during storage because phenolics can be oxidized or become less extractable after binding to proteins, so it should not be interpreted as evidence against their functional role [51].

The literature supports the hypothesis that low residual nitrite at day 7 corresponds with high antimicrobial activity because the nitrite is actively consumed to generate inhibitors. Sindelar & Milkowski [59] note that, for botulinal control in cured meat systems, the initially added nitrite level may be more influential than the residual nitrite measured during storage, suggesting that antimicrobial species formed via nitrite-related reactions could contribute substantially to inhibition. Furthermore, nitrites participate in the synthesis of highly reactive forms of nitric oxide (ONOO-/ONOOH) in the meat matrix, which have the potential to damage bacterial cells [16]. Concurrently, added nitrite is progressively depleted through nitrogen oxide reactions, and residual nitrite typically declines during storage [59]. By day 14, R3 showed the lowest counts, followed by PC and B3, while NC remained higher. Overall, B3 and R3 showed better microbial control than NC during storage, with partial comparability to PC at day 7 and day 14.

Across storage, pH generally declined slightly from day 0 to day 14 in most treatments, with treatment-specific differences emerging over time. At day 0, pH values were highest in NC, PC, and B3, while R3 and R6 were slightly low. At day 7, PC, B3, and B6 maintained higher pH values (about 6.43 to 6.46) compared with NC, R3, and R6, suggesting a treatment effect during mid-storage. The observation that R3 and R6 had slightly lower initial pH values compared to the controls and beetroot treatments can be explained by a more acidic nature of fermented radish mixture powder (pH 3.81) compared to the fermented beetroot mixture powder (pH 4.4). Studies by Choi et al. and Hwang et al. [45,48] support the finding, which researchers investigating the addition of pre-converted nitrites from fermented vegetables (spinach, lettuce, celery, and red beet) to raw meat batters found the same trend. They reported that the pH of the meat treated with pre-converted extracts was significantly lower than the controls. The authors clearly attributed this to the fact that fermented vegetable extracts naturally have low pH values (e.g., their fermented red beet extract had a pH of 4.65) due to the organic acids produced by the starter cultures during the pre-conversion incubation. Because the fermented radish mixture powder was more acidic than the fermented beetroot mixture powder in the current study, it showed lower starting pH values. By day 14, B3 showed the lowest pH, whereas R6 showed the highest, and the remaining treatments clustered around 6.23 to 6.28. Overall, pH differences were modest. At the end of the storage period, samples B3 and B6 (beetroot-incorporated) exhibited a significant decline in pH values, reaching levels comparable to the NC. The low pH values may explain the sensory trade-offs observed on day 14, where these samples received lower scores for taste and flavor acceptance compared to the radish-treated group. In a refrigerated low-salt sausage system, increasing fermented red beet levels decreased sausage pH, and overall acceptability scores decreased as fermented red beet levels increased [28].

### 3.7. Chemical Compositions

The chemical composition of beef burgers treated with different levels (3% and 6%) of fermented radish and beetroot mixture powder across refrigerated storage (0, 7, and 14 d) is shown in Table 6. The chemical composition values varied significantly with treatment, storage time, and their interaction (*p* < 0.01), indicating a treatment-dependent storage response. Overall, the fermented radish and beetroot mixture changed the chemical composition during cold storage, with some treatments showing values comparable to or better than PC at specific time points, especially at day 7.

Across storage, no uniform trend was observed for moisture content, as some treatments increased moisture content at day 7 then decreased at day 14 (for example, PC and B3), while others remained relatively lower and more stable (R6 and B6). Among the natural alternatives, B3 and R3 showed higher moisture than NC at day 7, whereas R6 and B6 were the lowest and not significantly different from each other. The amount of added fermented radish and fermented beetroot mixture powders directly affects the moisture content of the burgers. Based on the observations, the higher concentration (6%) resulted in lower moisture content compared to the lower concentration (3%). Fermented vegetable mixtures were added by replacing an equivalent percentage of the added water, which increased total solids and reduced the moisture of treated burgers [29,44]. A study on beef patties incorporated with destoned olive cake powder showed that moisture content gradually decreased as the powder concentration increased (from 0% to 6%) due to the rise in total solid contents [29]. Similarly, increasing the levels of Argel leaf powder in camel patties significantly decreased the moisture content of the raw patties [44]. High concentrations of vegetable powders (6%) create a denser product with more total solids and less free water. As a result, it shows less drip loss and more stable weight changes than the high-moisture controls [32]. This aligns with the cooking yield, which was consistently highest in R6 and high in B6, and diameter shrinkage was lowest in R6 at all days.

Protein content changed over time in different ways, depending on the formulation used. On day 7, NC recorded the highest protein (24.85%), while B6 (23.18%) was statistically similar to PC (23.13%), and R6 (23.56%) was also close to PC but remained significantly different. In contrast, R3 (22.50%) and B3 (22.32%) were lower than both NC and PC on day 7. Ingle et al. [78] reported that increasing beetroot powder substitution in cookies (5 to 20%) increased crude protein from 7.39% to 9.12%. A similar trend was reported for cakes, where increasing beetroot powder incorporation up to 25% increased crude protein from 6.10% to 12.42% [79]. At day 7, the inverse pattern between moisture and protein suggests that differences in protein percentage largely reflect water retention rather than true changes in absolute protein. NC, which lacked vegetable powders, may have experienced higher moisture loss during refrigerated storage, producing an apparent increase in protein percentage via a concentration effect. This is consistent with its lower cooking yield and higher shrinkage relative to the fiber-containing treatments. Fogarasi et al. [80] reported a moisture-driven concentrating effect in control of Vienna sausages, where higher water loss during storage increased protein percentage from 15.02% to 16.47%, while sausages formulated with plant showed stable protein due to better moisture retention. Conversely, B3 maintained the highest moisture, which likely diluted the protein percentage.

Fat content showed a treatment-dependent pattern during storage. PC and R3 increased progressively from day 0 to day 14, whereas B3 started with the highest fat percentage at day 0 and decreased thereafter. At day 7, R6 (8.16%) was the highest, followed by PC (7.85%), while R3 and B3 were identical (7.60%) and both exceeded NC (6.93%). B6 (6.39%) was lower than NC at day 7. Because proximate fat is reported on a wet weight basis, shifts in water retention can influence the measured fat percentage, such that higher values may partially reflect concentration effects rather than true fat gain. In fermented goat meat sausages, the reported fat percentage increased from 8.23% to 9.68% at day 0 to 19.83% to 21.87% after 30 days, and the authors attributed this rise to moisture loss during drying, causing a concentration effect on remaining components [81]. At day 7, R6 combined the highest fat percentage with the highest TBARS (Table 3), which is consistent with the role of lipids as oxidation substrates and the contribution of heme iron and myoglobin-mediated pathways to propagate lipid oxidation in comminuted meat systems [82]. However, this alignment was not consistent across all treatments and time points, indicating that oxidation was not governed by fat percentage alone. Notably, PC maintained the lowest TBARS at day 7 despite relatively high fat, which agrees with the antioxidant function of nitrite in cured meats. This effect is commonly attributed to nitrite-derived NO binding to and stabilizing heme iron, reducing iron-catalyzed initiation, and to NO or nitrosyl species quenching lipid radicals, thereby slowing chain oxidation [83].

Ash content was also significantly affected by treatment, storage, and their interaction, but within a narrower range. Some treatments remained relatively stable (NC, R6), while PC increased by day 14 (2.03%) and B3/B6 decreased to the lowest values by day 14 (1.34% and 1.37%, respectively). At day 7, NC and PC were similar (both about 1.70), B6 was comparable, while R3 and B3 were higher, and R6 was lower. Overall, the chemical composition of natural treatments showed improvements over NC and partial comparability with PC, especially at day 7, but no single treatment consistently matched PC across all proximate parameters [83,84].

### 3.8. Cooking Properties

Table 7 shows that cooking yield and diameter shrinkage were significantly affected by treatment, storage, and treatment x storage interaction (*p* < 0.01). Overall, radish and beetroot treatments, especially R6 and B6, improved cooking performance during cold storage compared with NC and PC. Cooking yield was the highest in R6 and high in B6, and diameter shrinkage was lowest in R6 at all days, indicating better water and fat retention during heating. This aligns with the idea that radish and beet ingredients can change water binding through solids, fiber, and protein network interactions, improving dimensional stability even if the proximate moisture percentage is not always highest [85].

Across storage, cooking yield declined at day 7, then partially recovered by day 14 in most treatments. At day 7, natural treatments showed higher cooking yield than both NC and PC, with R6 and B6 among the highest, followed by R3 and B3. By day 14, PC showed the lowest cooking yield, followed by NC, whereas R6 and B6 showed high values, followed by R3 and B3, indicating better cooking stability of these formulations. At day 7, cooking yield increased in R6 and B6 compared to NC and PC, and this improvement occurred together with the lowest diameter shrinkage in B6 and R6 versus NC and PC. Proximate moisture (Table 6) did not explain the higher yield, because at day 7 both R6 and B6 had the lowest moisture content, whereas B3 had the highest moisture but a lower cooking yield than R6 and B6. This indicates that dietary fibers could be the key factor in improving cooking yield. A study incorporating beetroot powder into chicken sausages found that the cooking yield and emulsion stability significantly improved because the dietary fibers in the beetroot powder acted to prevent moisture migration during the cooking process [52]. Similarly, research on pork patties formulated with dried radish leaves and roots found that cooking losses were significantly reduced in the treated groups. The authors concluded that the high water-binding capacity and moisture absorption of the radish dietary fibers improved the structural holding capacity, explaining why the treated patties held onto their mass better during cooking [86]. Across storage, diameter shrinkage tended to be lowest at day 7 and significantly increased at day 14 in all treatments. At day 7, natural treatments showed lower shrinkage than NC and PC, with B6 and R6 showing the lowest values, suggesting better dimensional stability during mid-storage. By day 14, R6 maintained the lowest shrinkage among all treatments, while NC, PC, B3, and B6 showed the highest shrinkage levels, and R3 remained intermediate. The lower shrinkage in the natural treatment shows that plant powders, which are rich in dietary fibers, possess excellent water and fat-absorbing capacities. When added at a high concentration (6%), these fibers absorb the free moisture and melted fat, causing them to swell. This swelling enables the plant fibers to interact tightly with the meat protein matrix, creating a preventive barrier against moisture and fat migration [29,44].

### 3.9. Texture Analysis Profile (TPA)

Table 8 shows that texture parameters were significantly affected by treatment, and most traits also showed a significant treatment x storage interaction (*p* < 0.05 and *p* < 0.01). Hardness and chewiness showed the clearest treatment separation during storage, where R3 produced the firmest texture at day 7 (hardness 0.41, chewiness 0.19), while NC and B3 increased markedly by day 14 (hardness 0.69 and 0.48, chewiness 0.33 and 0.18, respectively), whereas PC remained among the lowest at day 14 (hardness 0.10, chewiness 0.04).

At day 14, NC showed the highest hardness (0.69) and chewiness (0.33), and this coincided with the greatest diameter shrinkage (39.03%) and a relatively low to moderate cooking yield (55.42%). Collectively, these outcomes indicate increased cooking losses and a denser protein matrix. This interpretation aligns with the elevated oxidative status of NC (Table 3) and the practical observation of exudate and off-odor in the NC burger. Oxidative reactions and their secondary products can promote protein modifications that reduce water-holding capacity and favor tighter network formation upon heating, thereby increasing shrinkage and yield losses. For B3, hardness and chewiness increased by day 14 (0.48 and 0.18), and B3 also showed high shrinkage (37.88), which supports matrix tightening and moisture expulsion during heating, even though cooking yield remained slightly higher than NC (60.01). Because B3 had low TBARS at day 14 (Table 3), its firmness is less likely to be driven by oxidative toughening and more likely reflects a combination of plant solids and the lowest pH at day 14 (6.15), reducing water holding, together producing a firmer bite despite antioxidant protection. This cross-table linkage is also reinforced by R6 and B6, which had the highest yields and the lowest shrinkage at day 7, and remained relatively low in hardness at day 14, matching the expected inverse relationship between water-holding capacity-driven cooking performance and texture firming. A study evaluating the texture profile of beef burgers formulated with antioxidant-rich moringa powders during cold storage found that the hardness and chewiness of the raw meat patties significantly increased as the storage time progressed [32]. They explained that the progressive toughening over time is caused by moisture loss, changes in the protein structure, and increased friction and binding among the meat particles and the added plant fibers. This supports the observation that NC and B3 treatments became markedly harder and chewier at the end of the 14-day storage period [32].

Cohesiveness showed smaller changes than hardness, but beetroot treatments tended to reduce cohesiveness at day 7 (B3 0.75, B6 0.76) compared with NC and R3 (0.91 and 0.93). Springiness was comparatively stable across storage (storage effect not significant), with only modest shifts between treatments (about 0.40 to 0.50). Adhesion was treatment dependent, being highest in PC at day 0 (0.34) but increasing sharply in NC at day 14 (0.75). R6 and B6 showed lower adhesion compared with other treatments. Adhesion or stickiness in processed meat systems is influenced by the extraction of myofibrillar proteins, which contribute to particle binding and gel network formation. When a high concentration of plant powders (like 6%) is added, it introduces additional plant-derived solids, dietary fibers, and carbohydrates. These plant particles may dilute the meat proteins and interrupt the cross-linking of the protein network, preventing the batter from forming a highly sticky/adhesive structure. Akcan et al. [87] reported that adding corn flour (3%, 6%, 9%) to beef meatballs increased hardness while reducing adhesiveness versus the control, supporting that added non-meat solids disrupt the continuity of the myofibrillar protein network and lower batter stickiness.

Overall, R3 mainly increased firmness and chewiness at mid-storage, B3 contributed to higher firmness by the end of storage, and a higher percentage (6%) of vegetables added helped reduce stickiness, indicating that radish and beetroot formulations can modulate key texture attributes during cold storage.

### 3.10. Sensory Analysis

Table 9 shows that all sensory attributes were significantly affected by treatment, storage, and treatment x storage interaction (*p* < 0.01). Overall, radish and beetroot treatments maintained higher sensory scores than PC during cold storage.

Across storage, sensory scores generally declined in NC and PC, while radish and beetroot treatments retained higher scores, indicating better sensory stability over time. PC (nitrite-added) received lower sensory scores. NC was not evaluated at day 14 due to an unpleasant odor and excessive exudate during cooking, despite an acceptable microbial load. Off-odor can be driven by physicochemical deterioration, such as purge formation and oxidative off-odors, especially since TBARS in NC was high, and this may be associated with rancid odors in meat products.

At day 7, R3 achieved the highest scores across key attributes, including color, taste, texture, flavor, tenderness, and overall acceptability, outperforming PC and the remaining natural treatments. R6 also maintained high scores at day 7 and remained close to R3 for several attributes. Overall, sensory responses aligned with the mechanistic trends in color stability, lipid oxidation, and microbial indicators. R3 was the most consistent “balanced performer,” achieving top day 7 scores across attributes and retaining the highest overall acceptability at day 7 and 14, consistent with its high phenolics and DPPH inhibition at day 7, low TBARS at day 14, good microbial control, and favorable color retention.

In a study, the researchers formulated beef burgers with 2%, 4%, and 6% clove powder. Similar to R3 results, they found that burgers prepared with 2% and 4% concentrations retained the highest overall acceptability at the end of the storage period [71]. Also, investigating beef patties with 2%, 4%, and 6% destoned olive cake powder, the authors found that concentrations up to 4% maintained excellent sensory attributes throughout storage [29].

On the other hand, R6 has better color stability, tenderness, and cooking performance, but its consistently high TBARS signals a meaningful trade-off; it may protect appearance and yield while still allowing lipid oxidation pathways to progress. A comprehensive review of plant extracts in meat products highlights that choosing the optimal quantity of a plant extract is the major difficulty in meat reformulation [7]. Studies on extracts like barberry demonstrated that while moderate amounts successfully inhibit oxidation, adding higher quantities often fails to decrease TBARS values over extended storage [7].

By day 14, R3 remained the highest for overall acceptability and texture, while R6 showed the highest tenderness and good color stability, indicating that radish-based treatments were the most consistent performers at late storage. Beetroot treatments showed intermediate performance, where B6 generally retained higher taste and overall acceptability than B3 at day 14, while both maintained acceptable color scores. B3 and B6 showed moderate performance, while maintaining other quality parameters; they showed notable trade-offs regarding taste and flavor profiles towards the end of the storage period. B6 provided good protection against lipid oxidation, showing the lowest TBARS at day 14, together with a high DPPH inhibition at the same time point. However, its sensory scores were moderate compared with R3, suggesting that while B6 improved oxidative stability and helped preserve quality, beet-derived compounds may have introduced flavor or aroma notes that limited overall liking. Beetroot can contribute earthy or distinctive aroma notes, largely associated with geosmin and related volatiles, which can influence liking depending on dose and matrix [88]. The researchers tested 1.5%, 3.0%, and 4.5% beetroot powder in chicken sausages. They found that the highest concentration (4.5%) yielded the significantly lowest TBARS values and the highest DPPH radical inhibition throughout the 12-day storage period, which the authors linked to the greater contribution of beetroot-derived bioactive compounds, including phenolics and betalains. Despite the 4.5% treatment providing the absolute best protection against lipid oxidation, it received lower sensory scores for color, appearance, and overall acceptability compared to the moderate 3.0% treatment. The researchers concluded that while the highest dose optimized chemical stability, the 3.0% treatment was the ultimate “balanced performer” that maintained better overall sensory acceptability [52].

Overall, sensory results support R3, followed by R6, as the most promising natural alternatives for maintaining beef burger sensory quality during cold storage.

## 4. Conclusions

This study demonstrated the potential of radish and beetroot fermented powders, used as whole matrices without separating the converted fraction, as natural sources of nitrite and bioactive compounds partially improving quality attributes and supporting quality preservation of raw beef burgers during refrigerated storage. The natural treatments, particularly at day 7, improved color attributes, reduced lipid oxidation, and helped control microbial growth compared with the negative control, while showing performance that was in some cases close to that of the sodium nitrite treatment across most evaluated parameters. They also provided additional advantages, including higher cooking yield, greater phenolic content, good DPPH radical scavenging activity, better sensory acceptance, and lower diameter shrinkage. Among the natural treatments, radish generally showed better overall performance, whereas beetroot showed greater effectiveness in reducing TBARS at day 7. Notably, R3 showed the most favorable treatment performance when color, oxidative stability, microbial control, and sensory quality were considered together. These findings support the potential of radish fermented powders as a promising clean-label alternative that may reduce reliance on synthetic sodium nitrite in refrigerated meat products. However, the natural treatments did not fully match sodium nitrite under all conditions, and the results are limited to a laboratory-scale raw beef burger system under refrigerated storage using specific fermented powder levels. Further studies are needed to validate their performance under commercial processing conditions and in other meat products.

## Figures and Tables

**Table 1:** Formulation (%) of beef burgers with varied levels of fermented radish and beetroot mixture powder.

Ingredients (%)	Sample Groups					
	Control		Beetroot		Radish	
	NC	PC	B3	B6	R3	R6
lean meat	76.70	76.70	76.70	76.70	76.70	76.70
added fat	9	9	9	9	9	9
cold water	10.50	10.25	7.50	4.50	7.50	4.50
salt	1.20	1.20	1.20	1.20	1.20	1.20
White pepper	0.20	0.20	0.20	0.20	0.20	0.20
Black pepper	0.20	0.20	0.20	0.20	0.20	0.20
Garlic powder	0.20	0.20	0.20	0.20	0.20	0.20
Onion powder	2	2	2	2	2	2
Curing mixture (6.25% sodium nitrite + 93.75% NaCI)	0	0.25	0	0	0	0
Sodium ascorbate	0.05	0.05	0.05	0.05	0.05	0.05
Fermented beetroot mixture powder	0	0	3	6	0	0
Fermented radish mixture powder	0	0	0	0	3	6

NC; negative control, PC; positive control (nitrite), R3; 3% radish, R6; 6% radish, B3; 3% beetroot, B6; 6% beetroot. In the PC treatment, the curing premix consisted of 6.25% sodium nitrite and 93.75% NaCl. The premix was added at 0.25% of the formulation, corresponding to an ingoing sodium nitrite level of 156 mg/kg product.

**Table 2:** Nitrate and nitrite content, pH, and antioxidant activity (DPPH % inhibition) of radish and beetroot powders and their fermented powders.

Samples	No_3_ Nitrate Con. (mg/Kg)	No_2_ Nitrite Con. (mg/Kg)	pH	DPPH %
Beetroot powder	3805.42	<LOD	6.56	NA
Fermented beetroot	351.06	751.99	4.40	67.56
Radish powder	25,623.60	<LOD	6.16	NA
Fermented radish	1588.72	389.11	3.81	63.36

Values are means of triplicate samples. LOD: limit of detection. NA: not analyzed. fermentation or nitrate-to-nitrite pre-conversion incubated for 24 h at 30 °C.

**Table 3:** Residual nitrite contents, phenolic content, antioxidant activity (DPPH % inhibition), and lipid oxidation (TBARS) of beef burger treated with different levels (3% and 6%) of fermented radish and beetroot mixture powder during cold storage (4 °C) for 14 days.

Parameter	Storage	Treatments						*P_t_*	*P_s_*	*P_t×s_*
		NC	PC	R3	R6	B3	B6			
Residual NO_2_ (mg/kg)	0	1.40 ± 0.00 ^L^	32.48 ± 0.04 ^a^	1.65 ± 0.04 ^j^	4.57 ± 0.00 ^c^	3.84 ± 0.04 ^e^	2.89 ± 0.07 ^g^	**	**	**
	7	0.67 ± 0.04 ^n^	15.39 ± 0.00 ^b^	1.40 ± 0.00 ^L^	2.49 ± 0.00 ^h^	1.29 ± 0.00 ^m^	1.51 ± 0.00 ^k^			
	14	1.22 ± 0.04 ^m^	4.28 ± 0.04 ^d^	2.31 ± 0.04 ^i^	1.65 ± 0.04 ^j^	3.29 ± 0.04 ^f^	3.33 ± 0.04 ^f^			
Phenolic content (mg GAE/kg sample)	0	285.77 ± 0.39 ^m^	314.00 ± 0.00 ^j^	354.33 ± 0.33 ^d^	363.00 ± 0.00 ^c^	364.00 ± 0.00 ^b^	319.00 ± 0.00 ^h^	**	**	**
	7	264.50 ± 0.29 ^n^	312.00 ± 0.00 ^k^	447.00 ± 0.00 ^a^	350.50 ± 0.50 ^e^	262.00 ± 0.00 ^o^	330.00 ± 0.00 ^f^			
	14	34.54 ± 0.03 ^r^	37.43 ± 0.01 ^q^	328.00 ± 0.00 ^g^	317.00 ± 0.00 ^i^	128.67 ± 0.88 ^p^	300.33 ± 0.33 ^L^			
DPPH (% inhibition)	0	42.37 ± 0.00 ^m^	46.18 ± 0.00 ^k^	56.87 ± 0.22 ^e^	43.51 ± 0.00 ^L^	55.34 ± 0.44 ^f^	59.92 ± 0.00 ^d^	**	**	**
	7	42.75 ± 0.00 ^m^	33.97 ± 0.00 ^n^	48.86 ± 0.22 ^i^	25.57 ± 0.00 ^p^	53.44 ± 0.58 ^g^	46.95 ± 0.00 ^j^			
	14	49.62 ± 0.00 ^h^	24.81 ± 0.22 ^q^	30.15 ± 0.00 ^o^	69.85 ± 0.00 ^c^	72.90 ± 0.44 ^b^	76.34 ± 0.22 ^a^			
TBARS (mg MDA equivalents/kg)	0	1.71 ± 0.00 ^d^	0.96 ± 0.00 ^gh^	1.75 ± 0.00 ^cd^	1.99 ± 0.00 ^bc^	1.39 ± 0.00 ^ef^	1.98 ± 0.00 ^bc^	**	**	**
	7	2.26 ± 0.00 ^a^	0.74 ± 0.00 ^h^	2.22 ± 0.00 ^ab^	2.46 ± 0.00 ^a^	1.62 ± 0.00 ^de^	1.63 ± 0.00 ^de^			
	14	2.19 ± 0.00 ^ab^	1.28 ± 0.00 ^f^	1.12 ± 0.00 ^fg^	2.42 ± 0.00 ^a^	1.12 ± 0.00 ^fg^	0.89 ± 0.37 ^gh^			

Values are means of triplicate samples (±SE). Means not sharing a common superscript in columns and rows for each parameter are significantly different (*p* < 0.05) as assessed by Duncan’s multiple range test. NC; negative control, PC; positive control (nitrite), R3; 3% radish, R6; 6% radish, B3; 3% beetroot, B6; 6% beetroot, Pt; treatment, Ps; storage, Ptxs; treatment vs. storage. ** *p* < 0.01.

**Table 4:** Color properties of beef burger treated with different levels (3% and 6%) of fermented radish and beetroot mixture powder during cold storage (4 °C) for 14 days.

Parameter	Storage	Treatments						*P_t_*	*P_s_*	*P_txs_*
		NC	PC	R3	R6	B3	B6			
L*	0	43.91 ± 0.45 ^bc^	45.88 ± 1.19 ^ab^	45.10 ± 0.65 ^bc^	46.07 ± 0.74 ^ab^	35.51 ± 0.23 ^f^	31.20 ± 0.46 ^gh^	**	**	**
	7	42.74 ± 0.52 ^cd^	40.66 ± 0.61 ^de^	39.26 ± 0.05 ^e^	42.69 ± 0.80 ^cd^	34.23 ± 1.36 ^f^	29.45 ± 0.20 ^h^			
	14	45.54 ± 0.86 ^b^	48.43 ± 0.88 ^a^	43.99 ± 1.04 ^bc^	45.16 ± 1.09 ^bc^	40.98 ± 1.50 ^de^	33.16 ± 0.98 ^fg^			
a*	0	10.64 ± 0.11 ^a^	8.72 ± 0.73 ^c^	9.78 ± 0.15 ^b^	8.90 ± 0.18 ^c^	4.22 ± 0.04 ^gh^	7.71 ± 0.52 ^d^	**	**	**
	7	4.48 ± 0.08 ^fgh^	7.53 ± 0.11 ^d^	6.42 ± 0.20 ^e^	6.82 ± 0.28 ^de^	3.12 ± 0.17 ^ij^	4.65 ± 0.24 ^fgh^			
	14	2.84 ± 0.40 ^j^	2.63 ± 0.16 ^j^	5.37 ± 0.26 ^f^	6.98 ± 0.37 ^de^	3.92 ± 0.18 ^hi^	4.88 ± 0.07 ^fg^			
b*	0	12.99 ± 0.52 ^abc^	13.51 ± 0.32 ^a^	12.55 ± 0.06 ^abcd^	13.70 ± 0.49 ^a^	10.90 ± 0.01 ^efgh^	13.22 ± 0.51 ^ab^	**	**	**
	7	10.12 ± 0.42 ^fghi^	8.92 ± 0.32 ^i^	10.60 ± 0.40 ^efgh^	11.49 ± 0.72 ^cdef^	9.51 ± 0.36 ^ghi^	11.31 ± 0.16 ^def^			
	14	11.08 ± 0.49 ^defg^	11.84 ± 0.59 ^bcde^	9.68 ± 0.58 ^ghi^	10.07 ± 0.34 ^fghi^	9.30 ± 0.91 ^hi^	11.61 ± 0.76 ^cdef^			

Values are means of triplicate samples (±SE). Means not sharing a common superscript in columns and rows for each parameter are significantly different (*p* < 0.05) as assessed by Duncan’s multiple range test. NC; negative control, PC; positive control (nitrite), R3; 3% radish, R6; 6% radish, B3; 3% beetroot, B6; 6% beetroot, Pt; treatment, Ps; storage, Ptxs; treatment vs. storage. ** *p* < 0.01.

**Table 5:** Microbiological load and pH of beef burger treated with different levels (3% and 6%) of fermented radish and beetroot mixture powder during cold storage (4 °C) for 14 days.

Parameter	Storage	Treatments						*P_t_*	*P_s_*	*P_txs_*
		NC	PC	R3	R6	B3	B6			
Log 10 (CFU/g)	0	5.09 ± 0.05 ^gh^	4.39 ± 0.21 ^j^	4.86 ± 0.09 ^hi^	5.10 ± 0.08 ^gh^	5.04 ± 0.02 ^gh^	4.70 ± 0.00 ^i^	**	**	**
	7	6.23 ± 0.01 ^a^	5.82 ± 0.12 ^cd^	5.95 ± 0.03 ^bc^	6.04 ± 0.00 ^abc^	5.65 ± 0.03 ^de^	6.10 ± 0.01 ^ab^			
	14	5.64 ± 0.09 ^de^	5.11 ± 0.04 ^gh^	4.94 ± 0.18 ^hi^	5.26 ± 0.05 ^fg^	5.23 ± 0.04 ^g^	5.50 ± 0.04 ^ef^			
pH	0	6.47 ± 0.02 ^a^	6.43 ± 0.00 ^a^	6.23 ± 0.01 ^f^	6.12 ± 0.04 ^g^	6.43 ± 0.02 ^a^	6.30 ± 0.02 ^bcd^	**	**	**
	7	6.29 ± 0.02 ^bcde^	6.46 ± 0.00 ^a^	6.34 ± 0.01 ^b^	6.25 ± 0.01 ^def^	6.45 ± 0.01 ^a^	6.43 ± 0.01 ^a^			
	14	6.23 ± 0.03 ^f^	6.26 ± 0.01 ^def^	6.28 ± 0.01 ^cdef^	6.33 ± 0.03 ^bc^	6.15 ± 0.01 ^g^	6.24 ± 0.02 ^ef^			

Values are means of triplicate samples (±SE). Means not sharing a common superscript in columns and rows for each parameter are significantly different (*p* < 0.05) as assessed by Duncan’s multiple range test. NC; negative control, PC; positive control (nitrite), R3; 3% radish, R6; 6% radish, B3; 3% beetroot, B6; 6% beetroot, Pt; treatment, Ps; storage, Ptxs; treatment vs. storage; ** *p* < 0.01.

**Table 6:** Chemical composition (%) of beef burger treated with different levels (3% and 6%) of fermented radish and beetroot mixture powder during cold storage (4 °C) for 14 days.

Parameter	Storage	Treatments						*P_t_*	*P_s_*	*P_t×s_*
		NC	PC	R3	R6	B3	B6			
Moisture	0	78.27 ± 0.01 ^b^	67.83 ± 0.01 ^g^	57.78 ± 0.01 ^o^	65.24 ± 0.15 ^kL^	69.94 ± 0.01 ^f^	65.46 ± 0.01 ^jk^	**	**	**
	7	65.70 ± 0.00 ^j^	72.84 ± 0.00 ^c^	67.25 ± 0.15 ^h^	62.79 ± 0.57 ^n^	81.98 ± 0.05 ^a^	62.71 ± 0.00 ^n^			
	14	72.11 ± 0.01 ^d^	64.63 ± 0.00 ^m^	71.59 ± 0.03 ^e^	64.87 ± 0.18 ^lm^	66.68 ± 0.01 ^i^	65.15 ± 0.03 ^kL^			
Protein	0	22.24 ± 0.00 ^k^	23.01 ± 0.00 ^h^	20.81 ± 0.01 ^o^	24.77 ± 0.00 ^c^	22.98 ± 0.02 ^h^	24.01 ± 0.01 ^d^	**	**	**
	7	24.85 ± 0.00 ^b^	23.13 ± 0.01 ^g^	22.50 ± 0.00 ^i^	23.56 ± 0.04 ^f^	22.32 ± 0.02 ^j^	23.18 ± 0.01 ^g^			
	14	22.96 ± 0.04 ^h^	23.83 ± 0.02 ^e^	21.72 ± 0.01 ^m^	22.15 ± 0.03 ^L^	21.11 ± 0.01 ^n^	26.10 ± 0.05 ^a^			
Fat	0	6.23 ± 0.00 ^k^	6.23 ± 0.00 ^k^	6.23 ± 0.00 ^k^	7.63 ± 0.00 ^g^	9.85 ± 0.00 ^a^	7.70 ± 0.00 ^f^	**	**	**
	7	6.93 ± 0.01 ^i^	7.85 ± 0.00 ^e^	7.60 ± 0.01 ^g^	8.16 ± 0.03 ^d^	7.60 ± 0.01 ^g^	6.39 ± 0.00 ^j^			
	14	6.38 ± 0.02 ^j^	8.68 ± 0.02 ^c^	9.14 ± 0.01 ^b^	7.05 ± 0.01 ^h^	7.04 ± 0.00 ^h^	7.83 ± 0.03 ^e^			
Ash	0	1.74 ± 0.00 ^d^	1.92 ± 0.00 ^b^	1.81 ± 0.00 ^c^	1.50 ± 0.00 ^h^	1.70 ± 0.00 ^ef^	1.45 ± 0.00 ^i^	**	**	**
	7	1.70 ± 0.01 ^ef^	1.70 ± 0.00 ^ef^	1.81 ± 0.01 ^c^	1.50 ± 0.03 ^h^	1.81 ± 0.02 ^c^	1.69 ± 0.00 ^f^			
	14	1.73 ± 0.00 ^de^	2.03 ± 0.02 ^a^	1.62 ± 0.02 ^g^	1.64 ± 0.00 ^g^	1.34 ± 0.00 ^k^	1.37 ± 0.01 ^j^			

Values are means of triplicate samples (±SE). Means not sharing a common superscript in columns and rows for each parameter are significantly different (*p* < 0.05) as assessed by Duncan’s multiple range test. NC; negative control, PC; positive control (nitrite), R3; 3% radish, R6; 6% radish, B3; 3% beetroot, B6; 6% beetroot, Pt; treatment, Ps; storage, Ptxs; treatment vs. storage; ** *p* < 0.01.

**Table 7:** Cooking properties (%) of beef burger treated with different levels (3% and 6%) of fermented radish and beetroot mixture powder during cold storage (4 °C) for 14 days.

Parameter	Storage	Treatments						*P_t_*	*P_s_*	*P_t×s_*
		NC	PC	R3	R6	B3	B6			
Cooking yield	0	58.33 ± 0.15 ^g^	59.32 ± 1.14 ^g^	64.49 ± 1.39 ^f^	85.07 ± 1.59 ^a^	67.22 ± 0.96 ^e^	74.13 ± 0.37 ^b^	**	**	**
	7	49.67 ± 0.16 ^i^	58.18 ± 0.56 ^g^	69.29 ± 0.96 ^de^	74.71 ± 0.74 ^b^	68.63 ± 0.60 ^e^	71.40 ± 1.32 ^cd^			
	14	55.42 ± 0.27 ^h^	46.74 ± 0.27 ^j^	64.83 ± 0.59 ^f^	85.05 ± 0.58 ^a^	60.01 ± 0.31 ^g^	71.75 ± 0.24 ^c^			
Diameter shrinkage	0	30.36 ± 0.88 ^bc^	29.12 ± 2.06 ^cd^	23.25 ± 1.23 ^efg^	16.76 ± 2.08 ^h^	27.85 ± 0.83 ^cde^	34.64 ± 3.66 ^ab^	**	**	**
	7	24.73 ± 1.12 ^def^	27.06 ± 1.73 ^cde^	20.48 ± 0.92 ^fgh^	18.03 ± 1.60 ^h^	19.21 ± 1.42 ^gh^	17.08 ± 1.89 ^h^			
	14	39.03 ± 1.10 ^a^	38.21 ± 0.14 ^a^	34.76 ± 1.54 ^ab^	27.64 ± 0.21 ^cde^	37.88 ± 0.44 ^a^	36.49 ± 2.60 ^a^			

Values are means of triplicate samples (±SE). Means not sharing a common superscript in columns and rows for each parameter are significantly different (*p* < 0.05) as assessed by Duncan’s multiple range test. NC; negative control, PC; positive control (nitrite), R3; 3% radish, R6; 6% radish, B3; 3% beetroot, B6; 6% beetroot, Pt; treatment, Ps; storage, Ptxs; treatment vs. storage; ** *p* < 0.01.

**Table 8:** Texture analysis profile (TPA) of beef burger treated with different levels (3% and 6%) of fermented radish and beetroot mixture powder during cold storage (4 °C) for 14 days.

Parameter	Storage	Treatments						*P_t_*	*P_s_*	*P_t×s_*
		NC	PC	R3	R6	B3	B6			
Hardness (Kg)	0	0.05 ± 0.00 ^k^	0.29 ± 0.00 ^d^	0.09 ± 0.00 ^ijk^	0.11 ± 0.01 ^ghijk^	0.16 ± 0.03 ^efgh^	0.08 ± 0.01 ^jk^	**	**	**
	7	0.15 ± 0.01 ^efgh^	0.14 ± 0.01 ^efghi^	0.41 ± 0.03 ^c^	0.12 ± 0.01 ^fghij^	0.13 ± 0.01 ^fghij^	0.19 ± 0.01 ^e^			
	14	0.69 ± 0.04 ^a^	0.10 ± 0.01 ^hijk^	0.17 ± 0.02 ^ef^	0.19 ± 0.02 ^e^	0.48 ± 0.04 ^b^	0.17 ± 0.02 ^efg^			
Cohesiveness	0	0.89 ± 0.03 ^abcd^	0.93 ± 0.02 ^abc^	0.90 ± 0.02 ^abcd^	0.92 ± 0.02 ^abc^	0.81 ± 0.04 ^def^	0.90 ± 0.00 ^abcd^	**	*	**
	7	0.91 ± 0.01 ^abc^	0.83 ± 0.07 ^cdef^	0.93 ± 0.01 ^ab^	0.87 ± 0.02 ^abcd^	0.75 ± 0.04 ^f^	0.76 ± 0.04 ^ef^			
	14	0.95 ± 0.01 ^a^	0.90 ± 0.04 ^abcd^	0.84 ± 0.02 ^bcde^	0.86 ± 0.03 ^abcd^	0.88 ± 0.01 ^abcd^	0.87 ± 0.01 ^abcd^			
Springiness (mm)	0	0.47 ± 0.03 ^ab^	0.50 ± 0.00 ^a^	0.47 ± 0.03 ^ab^	0.43 ± 0.03 ^ab^	0.40 ± 0.00 ^b^	0.50 ± 0.00 ^a^	**	ns	*
	7	0.50 ± 0.00 ^a^	0.43 ± 0.03 ^ab^	0.50 ± 0.00 ^a^	0.43 ± 0.03 ^ab^	0.40 ± 0.00 ^b^	0.40 ± 0.00 ^b^			
	14	0.50 ± 0.00 ^a^	0.47 ± 0.03 ^ab^	0.40 ± 0.00 ^b^	0.43 ± 0.03 ^ab^	0.43 ± 0.03 ^ab^	0.43 ± 0.03 ^ab^			
Adhesion (mJ)	0	0.04 ± 0.01 ^gh^	0.34 ± 0.03 ^b^	0.05 ± 0.01 ^fgh^	0.03 ± 0.00 ^h^	0.14 ± 0.02 ^de^	0.03 ± 0.00 ^h^	**	**	**
	7	0.11 ± 0.01 ^efg^	0.21 ± 0.05 ^c^	0.18 ± 0.01 ^cd^	0.03 ± 0.01 ^h^	0.07 ± 0.01 ^efgh^	0.10 ± 0.00 ^efgh^			
	14	0.75 ± 0.06 ^a^	0.12 ± 0.01 ^def^	0.09 ± 0.02 ^efgh^	0.05 ± 0.00 ^gh^	0.25 ± 0.02 ^c^	0.02 ± 0.00 ^h^			
Chewiness (mJ)	0	0.14 ± 0.00 ^c^	0.02 ± 0.00 ^e^	0.04 ± 0.00 ^de^	0.04 ± 0.00 ^de^	0.05 ± 0.01 ^de^	0.04 ± 0.00 ^de^	**	**	**
	7	0.07 ± 0.00 ^d^	0.05 ± 0.01 ^de^	0.19 ± 0.02 ^b^	0.05 ± 0.01 ^de^	0.04 ± 0.00 ^de^	0.06 ± 0.00 ^de^			
	14	0.33 ± 0.02 ^a^	0.04 ± 0.01 ^de^	0.06 ± 0.01 ^de^	0.07 ± 0.02 ^d^	0.18 ± 0.03 ^b^	0.07 ± 0.01 ^d^			

Values are means of triplicate samples (±SE). Means not sharing a common superscript in columns and rows for each parameter are significantly different (*p* < 0.05) as assessed by Duncan’s multiple range test. NC; negative control, PC; positive control (nitrite), R3; 3% radish, R6; 6% radish, B3; 3% beetroot, B6; 6% beetroot, Pt; treatment, Ps; storage, Ptxs; treatment vs. storage. ns—not significant; * *p* < 0.05; ** *p* < 0.01.

**Table 9:** Sensory analysis of beef burger treated with different levels (3% and 6%) of fermented radish and beetroot mixture powder during cold storage (4 °C) for 14 days.

Parameter	Storage	Treatments						*P_t_*	*P_s_*	*P_t×s_*
		NC	PC	R3	R6	B3	B6			
Color	0	6.00 ± 0.35 ^a^	4.72 ± 0.53 ^bc^	6.39 ± 0.24 ^a^	7.00 ± 0.44 ^a^	6.11 ± 0.53 ^a^	6.11 ± 0.84 ^a^	**	**	**
	7	5.89 ± 0.20 ^ab^	4.50 ± 0.69 ^c^	7.22 ± 0.11 ^a^	6.50 ± 0.00 ^a^	6.56 ± 0.22 ^a^	6.44 ± 0.55 ^a^			
	14	NA	4.56 ± 0.36 ^c^	6.11 ± 0.33 ^a^	6.43 ± 0.23 ^a^	6.50 ± 0.31 ^a^	6.33 ± 0.25 ^a^			
Taste	0	5.61 ± 0.56 ^abc^	6.06 ± 0.53 ^abc^	6.44 ± 0.31 ^ab^	6.89 ± 0.29 ^a^	6.28 ± 0.58 ^ab^	6.22 ± 0.65 ^ab^	**	**	**
	7	4.72 ± 0.29 ^cd^	5.61 ± 0.43 ^abc^	6.89 ± 0.15 ^a^	5.94 ± 0.24 ^abc^	5.94 ± 0.55 ^abc^	5.89 ± 0.40 ^abc^			
	14	NA	3.89 ± 0.11 ^d^	6.00 ± 0.50 ^abc^	5.23 ± 0.34 ^bcd^	4.11 ± 0.68 ^d^	5.11 ± 0.43 ^bcd^			
Texture	0	5.00 ± 0.44 ^bc^	5.39 ± 0.59 ^abc^	6.50 ± 0.42 ^ab^	6.72 ± 0.28 ^a^	6.28 ± 0.56 ^ab^	6.61 ± 0.62 ^ab^	**	**	**
	7	4.00 ± 0.67 ^c^	4.94 ± 0.29 ^bc^	6.44 ± 0.15 ^ab^	6.22 ± 0.24 ^ab^	5.33 ± 0.10 ^abc^	6.17 ± 0.44 ^ab^			
	14	NA	3.94 ± 0.78 ^c^	6.89 ± 0.34 ^a^	6.44 ± 0.80 ^ab^	5.39 ± 0.55 ^abc^	5.89 ± 0.58 ^ab^			
Flavor	0	5.83 ± 0.25 ^bcd^	6.17 ± 0.19 ^abc^	6.44 ± 0.48 ^abc^	7.28 ± 0.24 ^a^	6.94 ± 0.28 ^ab^	5.83 ± 0.82 ^bcd^	**	**	**
	7	4.83 ± 0.33 ^de^	5.72 ± 0.20 ^bcd^	7.00 ± 0.35 ^ab^	6.17 ± 0.29 ^abc^	6.33 ± 0.44 ^abc^	5.83 ± 0.42 ^bcd^			
	14	NA	4.17 ± 0.38 ^e^	6.33 ± 0.25 ^abc^	5.79 ± 0.33 ^bcd^	4.33 ± 0.17 ^e^	5.33 ± 0.75 ^cde^			
Tenderness	0	4.94 ± 0.63 ^defg^	4.83 ± 0.59 ^efg^	6.06 ± 0.22 ^bcdef^	7.83 ± 0.25 ^a^	6.22 ± 0.29 ^bcde^	6.60 ± 0.70 ^abc^	**	**	**
	7	3.61 ± 0.24 ^gh^	4.67 ± 0.35 ^fg^	7.11 ± 0.24 ^ab^	7.06 ± 0.39 ^ab^	5.33 ± 0.17 ^cdef^	6.22 ± 0.62 ^bcde^			
	14	NA	3.11 ± 0.63 ^h^	6.78 ± 0.53 ^abc^	7.08 ± 0.63 ^ab^	5.00 ± 0.73 ^defg^	6.44 ± 0.31 ^abcd^			
Overall Acceptability	0	5.67 ± 0.67 ^cde^	5.56 ± 0.39 ^cdef^	6.56 ± 0.20 ^abc^	7.56 ± 0.11 ^a^	6.50 ± 0.48 ^abcd^	6.11 ± 0.58 ^bcd^	**	**	**
	7	4.39 ± 0.34 ^fg^	5.22 ± 0.48 ^def^	7.28 ± 0.15 ^ab^	6.28 ± 0.15 ^bcd^	5.61 ± 0.24 ^cdef^	5.89 ± 0.40 ^cd^			
	14	NA	3.78 ± 0.39 ^g^	6.67 ± 0.17 ^abc^	5.69 ± 0.41 ^cde^	4.50 ± 0.17 ^efg^	5.61 ± 0.68 ^cdef^			

Values are means of sensory scores (±SE). Means not sharing a common superscript in columns and rows for each parameter are significantly different (*p* < 0.05) as assessed by Duncan’s multiple range test. NC; negative control, PC; positive control (nitrite), R3; 3% radish, R6; 6% radish, B3; 3% beetroot, B6; 6% beetroot, Pt; treatment, Ps; storage, Ptxs; treatment vs. storage; ** *p* < 0.01. NA: Not analyzed.

## Data Availability

The original contributions presented in this study are included in the article. Further inquiries can be directed to the corresponding author.
